# Role of 12 lead ECG Q-waves as a marker of myocardial infarction in the era of cardiac magnetic resonance

**DOI:** 10.1186/1532-429X-18-S1-P239

**Published:** 2016-01-27

**Authors:** Amardeep Ghosh Dastidar, Alexander Carpenter, Jonathan C Rodrigues, Catherine R Wilson, Samantha R Kestenbaum, Anna Baritussio, Alberto Palazzuoli, Angus K Nightingale, Andreas Baumbach, Chiara Bucciarelli-Ducci

**Affiliations:** grid.410421.20000000403807336NIHR Cardiovascular Biomedical Research Unit, Bristol Heart Institute, Bristol, United Kingdom

## Background

Q-waves on 12 lead ECG is considered a marker of a large and/or transmural myocardial infarction (MI). Late gadolinium enhancement (LGE) cardiovascular magnetic resonance (CMR) accurately identifies myocardial infarction and has become the gold standard for the assessment of myocardial viability. However, CMR is not universally available and clinicians often have to make assumptions based solely on presence/location of Q waves.

## Aim

To determine the diagnostic accuracy of Q-waves on 12 lead ECG to identify the presence and location of myocardial scarring. To ascertain the CMR predictors of Qwave.

## Methods

Data was collected on consecutive patients suspected ischaemic heart disease (from April 2013 to Mar 2014). Exclusion criteria-presence of any non-ischaemic heart disease that may cause Q-Wave. Pathological Q-waves - deflection > 25% of the subsequent R wave, or being > 40 ms in width and > 2 mm in amplitude in >1 corresponding lead. Q waves in any 2 or more precordial leads from V1-V4 reflected LAD territory. A comprehensive CMR protocol was used. Transmural infarction was defined as >50% LGE. Univariate and multivariate logistic regression analyses were performed to determine the relation between the presence/absence of Q waves and CMR variables.

## Results

498 patients were included (mean age of 64 ± 12 years, 71% males). 290 patients demonstrated MI, 157 were transmural and 133 sub-endocardial based on CMR LGE. The overall diagnostic accuracy of Q-wave as a marker of transmural MI was 66% and the diagnostic accuracy of Q waves as a predictor of previous MI (composite of sub-endocardial and transmural) was only 55%. 126 had pathological Q-waves on 12 lead ECG, 40% had LAD territory Q waves, 55% non-LAD and 5% a combination. Of those with LAD Q waves, 68% demonstrated LAD territory LGE and in non-LAD Q waves, 67% demonstrated a non-LAD territory infarct by LGE.

Univariate predictors of the presence of a Q wave included any LGE, spatial extent of scar tissue, transmurality(50 or 75% LGE), and total scar score. On multivariate analysis, total scar score and >75% thickness LGE remained significant for prediction of the presence of a Q wave on the ECG.

## Conclusions

Our study demonstrates that the presence of pathological Q-waves on 12 lead ECG is not only a poor marker of myocardial scarring, but also a poor predictor of viability when compared to CMR. The study also highlights presence or absence of a Q wave on the ECG correlates only with total scar score and >75% wall thickness LGE. Our study also demonstrates the limitation of Q-wave in localizing the coronary artery territory. In their clinical decision making process, clinicians needs to be aware of the limitations of ECG Q-waves.

**Table 1:** Predictors of ECG Q-wave

Parameter	Odds	95% CI Univariate analysis	p-value	Odds	95% CI Multivariate analysis	p-value
Age	1.0	.98-1.02	.706	1.0	.98-1.01	.976
Sex	1.23	.76-1.99	.393	0.86	.50-1.47	.592
LGE	2.99	1.84-4.84	<.0001	1.76	0.83-3.33	.147
>50% LGE	.343	0.22-0.58	<0.001	0.96	0.47-1.93	.899
>75% LGE	.533	0.30-0.92	.024 2.29	2.29	1.05-4.99	.037
No. of segments	1.19	1.11-1.26	<0.001	0.82	0.65-1.04	.104
Scar score	1.07	1.05-1.10	<0.001	1.15	1.04-1.27	.004

LGE -late gadolinium enhancement.

